# Mitotane Nanocarriers for the Treatment of Adrenocortical Carcinoma: Evaluation of Albumin-Stabilized Nanoparticles and Liposomes in a Preclinical In Vitro Study with 3D Spheroids

**DOI:** 10.3390/pharmaceutics14091891

**Published:** 2022-09-07

**Authors:** Carolin Langer, Monika Köll-Weber, Martin Holzer, Constanze Hantel, Regine Süss

**Affiliations:** 1 Institute of Pharmaceutical Sciences, Department of Pharmaceutical Technology and Biopharmacy, University of Freiburg, Sonnenstraße 5, 79104 Freiburg, Germany; 2 Department of Endocrinology, Diabetology and Clinical Nutrition, University Hospital Zurich, Wagistrasse 21, 8952 Schlieren, Switzerland

**Keywords:** mitotane, adrenocortical carcinoma, albumin nanoparticles, liposomes, cancer therapy, 3D spheroids, dual centrifugation

## Abstract

Adrenocortical carcinoma (ACC) is a heterogeneous malignancy related to poor prognosis and limited treatment options. The orphan drug mitotane (MT) is still a cornerstone in ACC therapy, however, its application is characterized by low aqueous solubility, poor bioavailability, and unfavorable pharmacokinetics, often resulting in below-target plasma concentrations or toxic side effects. Throughout the last decades, nanoparticulate formulations have become attractive carriers to improve anticancer therapy. In this study, injectable MT liposomes (DOPC-MT) and albumin-stabilized MT nanoparticles (BSA-MT) were investigated in depth with respect to their physicochemical properties, and their colloidal and therapeutical stability upon storage. Furthermore, in vitro cytotoxicity was evaluated using the ACC model cell line NCI-H295R for preparing multicellular tumor spheroids, and was compared to non-malignant human dermal fibroblasts. Our results clearly demonstrate that BSA-MT, unlike DOPC-MT, represents a stable and storable MT formulation with a high drug concentration in an aqueous medium. Dual centrifugation was established as a reproducible method for nanoparticle preparation. Although an efficient cytotoxic effect on ACC tumor spheroids was demonstrated, concomitant low toxicity to fibroblasts suggests that higher drug concentrations may be tolerated in vivo. Consequently, BSA-MT is a novel and promising therapeutical approach to address key challenges in MT treatment.

## 1. Introduction

Adrenocortical carcinoma (ACC) is a rare, heterogeneous tumor that is often associated with a dismal prognosis [1]. The aggressiveness of the cancer leads to an overall 5-year survival rate between 16% and 47% and radical resection still offers the best chance of cure [1,2]. However, a substantial number of patients experience recurrent disease [3]. Mitotane (MT) is a key drug for both adjuvant treatment after surgical resection and palliative therapy for advanced ACC. The adrenolytic drug is applied alone or in combination with the classic cytostatic drugs etoposide, doxorubicin, and cisplatin (EDP-M regimen) [4]. Endoplasmatic reticulum stress caused by inhibition of sterol-O-acyl transferase 1 has been shown to be a key molecular effect of MT; however, the exact mechanism of action is still incompletely understood [5]. In general, MT treatment is characterized by many challenges: To date, plasma MT concentrations above 14 mg/L (44 µM) are recommended to achieve a therapeutic response, whereas toxic side effects increase at concentrations above 20 mg/L (62 µM) [1,2,6]. Given such a narrow therapeutic window, therapeutic drug monitoring is indispensable. Furthermore, there is a pronounced interindividual variability in the pharmacokinetics of the orally administered drug. Even with doses of up to 6 g MT per day, a substantial number of patients do not reach target plasma concentrations [7]. This poor and variable absorption is presumably caused by the low aqueous solubility of MT [8]. In addition, it was shown that the majority of MT in serum is bound to lipoprotein fractions, whereas the lipoprotein-free drug has the most potent desired cytotoxic effects [9,10].

Nanotechnological formulations of chemotherapeutic drugs have been widely used to improve their therapeutic efficacy. Liposomes in particular have been found to be beneficial for improving the solubility of hydrophobic drugs, and are often described as circulating drug depots with the ability to accumulate in solid tumors [11,12,13]. Even though it is still under discussion if the enhanced permeability and retention effect is the driving force for this witnessed accumulation, several liposomes incorporating cytostatic drugs have already been approved and have moreover been associated with decreased side effects [13,14,15,16,17]. Due to its hydrophobic nature, MT could in principle also be incorporated into the lipid bilayer. However, MT causes a disturbance of bilayer structures, making it quite difficult to develop liposomal formulations with it. This membrane interaction was observed primarily in the presence of phosphatidylethanolamine and/or cholesterol [18]. For that reason, we have omitted these commonly used lipids and developed MT liposomes based on 1,2-dioleoyl-sn-glycero-3-phosphocholine (DOPC-MT), with a limited drug content of 10 mol%.

A second promising approach in tumor therapy is nanoparticles (NPs) stabilized by albumin. This physiological protein is an attractive carrier as it is biodegradable, biocompatible, and soluble in water [19]. A well-known example of an approved albumin-based nanoparticulate drug formulation is Abraxane^®^ (*nab*-Paclitaxel). In the phase III study leading to its approval, patients with metastatic breast cancer tolerated higher drug doses, had significantly higher response rates, and had longer times of tumor progression in comparison to the standard paclitaxel regimen [20]. Similar to MT, paclitaxel is highly protein-bound in plasma. However, the unbound fraction is pharmacologically active [21]. Interestingly, it has been observed that the application of *nab*-Paclitaxel led to an increased fraction of unbound drugs without an increase in toxicity [21]. With these promising results, *nab*-Paclitaxel was also investigated for the treatment of ACC in mouse xenograft models and has been shown to be more effective than MT in reducing tumor weight [22]. Based on the distinct improvement in paclitaxel tumor therapy, as well as the similarities in treatment challenges between paclitaxel and MT, we developed an albumin-stabilized MT formulation (BSA-MT). To do this, BSA-MT was prepared through dual centrifugation as a novel technique for enabling a closed vial system for cytostatic drug handling.

In the field of oncology, in particular, drug potency and selectivity are of crucial importance during formulation development and they are often pre-evaluated in 2D and 3D in vitro cell culture systems. Whereas 2D cell monolayers are frequently used as primary testing models both for cancer cells and non-malignant cells, there is growing evidence that these models are unable to imitate the physiological properties of in vivo tumors and their drug resistance [23,24,25]. The need for a more predictive and powerful in vitro system for later translation to tumor xenografts and clinical studies has led to the development of in vitro 3D tumor models. Multicellular tumor spheroids (MCTS) are one of the most commonly used 3D culturing approaches, mimicking micrometastases or intervascular regions of large solid tumors [26]. The similarity is achieved by an outer layer of rapidly proliferating cells, a middle layer of quiescent cells, and, depending on spheroid diameter, a core of necrotic cells [27]. Upon interaction with the 3D extracellular matrix present, the tumor cells re-establish a pattern of differentiation similar to that found in vivo, which furthermore affects gene and protein expression and resistance to anticancer drugs [24,26]. Thus, MCTS are a useful model to bridge the gap between 2D cell monolayer systems and xenografts, enabling the assessment of novel anticancer drug delivery systems.

In this study, the main challenges in MT therapy—the poor bioavailability and unfavorable pharmacokinetics—were addressed by developing two injectable NP formulations. Both the liposomal MT and the albumin-stabilized MT were characterized with regard to their physicochemical properties, and NP long-term stability was evaluated with a focus on particle size, drug content, and efficacy in ACC cell culture (NCI-H295R). In general, a novel anticancer formulation should affect the target tumor tissue, while not harming healthy tissues, leading to toxic side effects. For that reason, we evaluated the desired cytotoxic effect of DOPC-MT and BSA-MT in an ACC MCTS model, while ensuring tolerability in human dermal fibroblasts (GM00038). Based on the knowledge that higher doses of *nab*-Paclitaxel can be applied without increased side effects, we moreover tested higher concentrations of the NP formulations that we developed (64 mg/mL, 200 µM) compared to the standard MT regimen (14–20 mg/mL).

## 2. Materials and Methods

### 2.1. Materials

Bovine serum albumin (BSA) and sodium azide (NaN_3_) were purchased from Sigma-Aldrich Chemie GmbH (Taufkirchen, Germany). 1,2-dioleoyl-sn-glycero-3-phosphocholine (DOPC) was kindly supplied by Lipoid GmbH (Ludwigshafen, Germany). Mitotane, di-sodium hydrogen phosphate dihydrate (Na_2_HPO_4_ × 2 H_2_O), and sodium dihydrogen phosphate (NaH_2_PO_4_) were bought from Merck KGaA (Darmstadt, Germany). Sodium hydroxide (NaOH), sodium chloride (NaCl), sodium bicarbonate (NaHCO_3_) formic acid, hydrochloric acid (HCl, 25%), sucrose, and glutaraldehyde (25%) were obtained from Carl Roth GmbH & Co. KG (Karlsruhe, Germany). Methanol, ethanol, and acetonitrile were of HPLC grade and purchased from VWR International GmbH (Darmstadt, Germany). Coomassie Plus^®^ protein assay reagent was received from Thermo Fisher Scientific (Rockford, IL, USA). Water was purified using an Arium^®^ Pro system from Sartorius AG (Göttingen, Germany). Yttrium oxide-stabilized zirconium oxide beads (milling beads) in the size ranges of 0.3–0.4 mm and 1.4–1.6 mm were obtained from Sigmund Lindner GmbH (Warmensteinach, Germany). Dual centrifugation vials (conical screw cap vials, 2 mL) were purchased from Sarstedt AG & Co. KG (Nümbrecht, Germany). For cell culture experiments, Dulbecco’s Modified Eagle Medium F-12 Nutrient Mixture (DMEM/F12, 1:1), insulin-transferrin-selenium (ITS, 100×), calcein-acetoxymethyl ester (calcein-AM) and propidium iodide were obtained from Life Technologies Ltd. (Paisley, UK). Minimum Essential Medium Eagle powder and fetal calf serum (FCS) were bought from Sigma-Aldrich Chemie GmbH (Taufkirchen, Germany) and the serum substitute Ultroser^®^ G from Pall Biosepra (Cergy-Saint-Christophe, France). Dulbecco’s phosphate-buffered saline w/o Ca^2+^ and Mg^2+^ (PBS) and trypsin/EDTA 0.05%/0.02% were received from PAN-Biotech GmbH (Aidenbach, Germany). CellTiter-Glo^®^ luminescent cell viability assay and CellTiter-Glo^®^ 3D cell viability assay were from Promega GmbH (Walldorf, Germany). The human adrenocortical carcinoma cell line NCI-H295R was purchased from CLS Cell Lines Service GmbH (Eppelheim, Germany) and human dermal fibroblasts (GM00038) were received from Coriell Institute for Medical Research (Camden, NJ, USA).

Bovine serum albumin (BSA) and sodium azide (NaN_3_) were purchased from Sigma-Aldrich Chemie GmbH (Taufkirchen, Germany). 1,2-dioleoyl-sn-glycero-3-phosphocholine (DOPC) was kindly supplied by Lipoid GmbH (Ludwigshafen, Germany). Mitotane, di-sodium hydrogen phosphate dihydrate (Na_2_HPO_4_ × 2 H_2_O), and sodium dihydrogen phosphate (NaH_2_PO_4_) were bought from Merck KGaA (Darmstadt, Germany). Sodium hydroxide (NaOH), sodium chloride (NaCl), sodium bicarbonate (NaHCO_3_) formic acid, hydrochloric acid (HCl, 25%), sucrose, and glutaraldehyde (25%) were obtained from Carl Roth GmbH & Co. KG (Karlsruhe, Germany). Methanol, ethanol, and acetonitrile were of HPLC grade and purchased from VWR International GmbH (Darmstadt, Germany). Coomassie Plus^®^ protein assay reagent was received from Thermo Fisher Scientific (Rockford, IL, USA). Water was purified using an Arium^®^ Pro system from Sartorius AG (Göttingen, Germany). Yttrium oxide-stabilized zirconium oxide beads (milling beads) in the size ranges of 0.3–0.4 mm and 1.4–1.6 mm were obtained from Sigmund Lindner GmbH (Warmensteinach, Germany). Dual centrifugation vials (conical screw cap vials, 2 mL) were purchased from Sarstedt AG & Co. KG (Nümbrecht, Germany). For cell culture experiments, Dulbecco’s Modified Eagle Medium F-12 Nutrient Mixture (DMEM/F12, 1:1), insulin-transferrin-selenium (ITS, 100×), calcein-acetoxymethyl ester (calcein-AM) and propidium iodide were obtained from Life Technologies Ltd. (Paisley, UK). Minimum Essential Medium Eagle powder and fetal calf serum (FCS) were bought from Sigma-Aldrich Chemie GmbH (Taufkirchen, Germany) and the serum substitute Ultroser^®^ G from Pall Biosepra (Cergy-Saint-Christophe, France). Dulbecco’s phosphate-buffered saline w/o Ca^2+^ and Mg^2+^ (PBS) and trypsin/EDTA 0.05%/0.02% were received from PAN-Biotech GmbH (Aidenbach, Germany). CellTiter-Glo^®^ luminescent cell viability assay and CellTiter-Glo^®^ 3D cell viability assay were from Promega GmbH (Walldorf, Germany). The human adrenocortical carcinoma cell line NCI-H295R was purchased from CLS Cell Lines Service GmbH (Eppelheim, Germany) and human dermal fibroblasts (GM00038) were received from Coriell Institute for Medical Research (Camden, NJ, USA).

### 2.2. Nanoparticle Preparation

#### 2.2.1. Preparation of Albumin-Stabilized Mitotane (BSA-MT)

BSA (50 mg/mL) was dissolved in purified water, adjusted to pH 8.2, and filtered through a 0.20 µm cellulose mixed esters syringe filter (Macherey-Nagel, Düren, Germany). Mitotane was dissolved in ethanol (20 mg/mL). Aliquots of 1 mL BSA solution, 0.25 mL mitotane solution, and 0.6 g milling beads (0.3–0.4 mm) were added to dual centrifugation vials. Yttrium oxide-stabilized zirconium oxide beads were chosen as milling beads due to their high wear resistance and proven quality for pharmaceutical applications, in particular for the preparation of nanoscale products. For NP formation, the dual centrifuge (DC) ZentriMix 380 R (Andreas Hettich GmbH & Co. KG, Tuttlingen, Germany) was used. The samples were centrifuged at 2350 rpm and 15 °C for 30 min, followed by a cooling run at 200 rpm, 4 °C for 30 min. NPs were stabilized by crosslinking 40% of the BSA amino groups as previously described [28]. Therefore, 11.4 µL glutaraldehyde solution (8%, m/m) was added and the samples were incubated at 500 rpm and 22 °C for 24 h using a Thermomixer comfort (Eppendorf AG, Hamburg, Germany). The resulting NPs were purified by 3 cycles of differential centrifugation (12,000× *g*, 8 min, Centrifuge 5417R, Eppendorf AG, Hamburg, Germany) and redispersion of the pellet in 1.5 mL purified water using a Sonorex RK 100 ultrasonic bath (Bandelin electronic GmbH & Co. KG, Berlin, Germany) for 5 min. In the last cycle, the BSA-MT NPs were redispersed in 1.0 mL purified water.

#### 2.2.2. Preparation of Mitotane Liposomes (DOPC-MT)

Liposomes were also prepared by dual centrifugation as described by Massing et al. [29,30]. However, a new formulation was developed for the incorporation of mitotane: DOPC (23.6 mg/mL) and mitotane (20 mg/mL) were each dissolved in ethanol, and aliquots of 667 µL DOPC solution and 32 µL mitotane solution were transferred to dual centrifugation vials. The solvent was evaporated at 25 °C using the vacuum centrifuge Concentrator plus (Eppendorf AG, Hamburg, Germany) for 1.5 h. The resulting lipid films were dried for 2 h at 30 mbar using a rotavapor IKA RV 10 digital (VWR International GmbH, Darmstadt, Germany) to ensure complete evaporation of the solvent. For preparing the dispersion medium, sucrose (85.5 mg/mL) and NaCl (1.5 mg/mL) were dissolved in purified water (pH 7.4) and filtered through a 0.20 µm cellulose mixed esters syringe filter (Macherey-Nagel, Düren, Germany). The dispersion medium as aqueous phase (24 µL) and milling beads (0.4 g, 1.4–1.6 mm) were added to the lipid films and the vials were dual centrifuged (2350 rpm, 15 °C, 30 min) to produce highly concentrated, vesicular phospholipid gels [29]. These gels were further hydrated with 80 µL aqueous phase and centrifuged again for 5 min. The resulting liposomes were diluted to a volume of 1 mL with dispersion medium to obtain the final DOPC-MT NP.

For cell culture experiments, DOPC control liposomes were prepared similarly but without the addition of mitotane.

### 2.3. Nanoparticle Characterization

#### 2.3.1. Particle Size and Zeta Potential

The average hydrodynamic diameter and polydispersity index (PDI) of the NP formulations were analyzed by photon correlation spectroscopy (PCS) using a ZetaPlus from Brookhaven Instruments Corporation (Holtsville, NY, USA). For BSA-MT analysis, 5 µL of the sample was diluted with 1.995 mL purified water (filtered through a 0.20 µm filter). For DOPC-MT analysis, 10 µL of the sample was diluted with 990 µL dispersion medium (filtered through a 0.20 µm filter). All samples were equilibrated for 2 min in the ZetaPlus instrument to reach the measuring temperature of 25 °C. Each measurement consisted of 10 individual runs and was performed at a 90° angle. Zeta potential was determined with a Zetasizer Nano-ZS (Malvern Instruments, Worcestershire, UK) via laser Doppler microelectrophoresis at 25 °C.

#### 2.3.2. High-Performance Liquid Chromatography

Drug content, drug recovery, and drug-to-lipid ratio were analyzed by high-performance liquid chromatography coupled to a diode array detector and a corona-charged aerosol detector (HPLC-DAD-CAD) as previously described [31]. This method enables the simultaneous quantification of the drug and the spectrophotometrically inactive phospholipid. In brief, an Ultimate 3000 HPLC system (Thermo Fisher Scientific GmbH, Dreieich, Germany) in combination with a Luna C18(2) column (150 mm × 4.6 mm, particle size 3 µm, Phenomenex Ltd., Aschaffenburg, Germany) was used for chromatographic separation. The column was tempered to 35 °C and the DAD was set to a wavelength of 229 nm for mitotane detection. Calibration samples for mitotane (1–200 µg/mL) and DOPC (10–1000 µg/mL) dissolved in ethanol were used for quantification. Elution was initially performed with 20% (*v*/*v*) eluent A (acetonitrile/purified water/formic acid, 90:10:0.3, *v*/*v*/*v*), 70% (*v*/*v*) eluent B (methanol/formic acid, 100:0.3, *v*/*v*) and 10% (*v*/*v*) eluent C (water/formic acid, 100:0.3, *v*/*v*) for 4 min. In the following 2 min, eluent C was linearly decreased to 0%. The composition of 20% eluent A and 80% eluent B was kept constant for 18 min and then brought back to the initial state until the run was stopped after 26 min. For BSA-MT analysis, elution was performed isocratically with 20% eluent A, 70% eluent B, and 10% eluent C for 6 min. A flow rate of 1 mL/min and an injection volume of 10 µL were used for all experiments. Both BSA-MT and DOPC-MT were diluted 1:19 (*v*/*v*) to obtain mitotane and lipid concentrations within the calibration ranges. For this purpose, DOPC-MT was dissolved in ethanol for subsequent analysis, whereas BSA had to be removed prior to analysis by the addition of methanol and centrifugation (10,000× *g*, 4 °C, 15 min). The resulting supernatant was used for mitotane quantification. The yield of BSA-MT was calculated by the total amount of mitotane used for NP preparation (MT_total_) and the resulting amount of mitotane after NP purification (MT_purif_) (Equation (1)). In contrast to BSA-MT, a purification of DOPC-MT was not necessary. Thus, the drug-to-lipid ratio instead of the yield was chosen as a characterization parameter. The drug-to-lipid ratio was calculated by the concentrations of mitotane (MT_lip_) and DOPC (DOPC_lip_) as determined after liposome preparation (Equation (2)).

(1) $$\mathrm{BSA}-\mathrm{MT}\;\mathrm{yield}\;\left[\%\right]=\frac{{\mathrm{MT}}_{\mathrm{purif}}}{{\mathrm{MT}}_{\mathrm{total}}}\cdot 100\%$$

(2) $$\mathrm{MT}/\mathrm{DOPC}\;\left[\mathrm{mol}/\mathrm{mol}\right]=\frac{{\mathrm{MT}}_{\mathrm{lip}}}{{\mathrm{DOPC}}_{\mathrm{lip}}}\;\;$$

#### 2.3.3. Asymmetric Flow Field-Flow Fractionation (AF4)

Particle size distribution, the occurrence of free albumin, and particle stability during storage were all analyzed by AF4. The system consisted of a flow controller Eclipse AF4, a multi-angle light scattering (MALS) detector DAWN Heleos II (Wyatt Technology, Santa Barbara, CA, USA), and a UV detector 1260 Infinity G1314F (Agilent Technologies Inc., Santa Clara, CA, USA). For separation, an Eclipse Short Channel equipped with a regenerated cellulose membrane (10 kDa cutoff) and a 350 µm W-type spacer (Wyatt Technology, Santa Barbara, CA, USA) was used. All measurements were conducted in phosphate buffer pH 6.6 (1.44 g/L NaH_2_PO_4_, 0.90 g/L Na_2_HPO_4_ × 2 H_2_O, 8.77 g/L NaCl, 0.20 g/L NaN_3_) as mobile phase at a detector flow rate of 1.0 mL/min. The sequence was subdivided into 6 steps (Vx = cross flow in mL/min): (1) elution (2 min, Vx: 1.0); (2) focus (2 min, Vx: 1.0), focus + inject (3 min, Vx: 1.0, inject flow: 0.40 mL/min), repeated 3 times with a final focus step; (3) elution (30 min, linear Vx gradient: 0.50–0.02); (4) elution (20 min, Vx: 0.02); (5) elution (10 min, Vx: 0.0); (6) elution + inject (5 min, Vx: 0.0). Samples were diluted 1:2 (*v*/*v*) with purified water and injected with a volume of 10 µL. To achieve membrane saturation, the diluted samples were injected 9 times prior to first analysis. MALS detector signals from all 18 scattering angles were used to calculate the root mean square (rms) radii of the nanoparticles (ASTRA 8 software (Wyatt Technology, Santa Barbara, CA, USA), particles mode, Berry formalism).

### 2.4. Stability Analysis

#### 2.4.1. Storage Stability

For storage stability studies, both BSA-MT and DOPC-MT were stored at 4–8 °C and 20–24 °C for 6 months. After 0, 1, 7, 14, 28, 61, 91, and 183 days, the samples were characterized by measuring particle size, zeta potential, and mitotane content as described in Section 2.3.1. The drug content of all samples was given in percent of the initially measured amount. Furthermore, DOPC-MT NPs were filtered through a 0.22 µm polyvinylidene fluoride syringe filter (Merck Millipore Ltd., Carrigtwohill, Ireland) to separate the liposomes from leaked, insoluble mitotane. The drug-to-lipid ratio was calculated and related to the initial drug-to-lipid ratio after liposome preparation for obtaining drug retention.

Bradford assay was used to determine the amount of detached albumin from BSA-MT during storage at 4–8 °C. Directly after NP preparation (0 days) and 3 months (91 days) of storage, 250 µL of the samples were centrifuged at 16,000× *g* and 4 °C for 20 min. Aliquots (50 µL) of the supernatant were incubated with 250 µL Coomassie Plus^®^ protein assay reagent in a 96-well microtiter plate for 10 min and subsequently analyzed with a SpectraCount^®^ multi-well spectrophotometer (Packard Instrument Company Inc., Downers Grove, IL, USA) at a wavelength of 590 nm. A concentration range of 15–500 µg/mL BSA in purified water was used for calibration.

Furthermore, BSA-MT stability was analyzed by AF4 as described in Section 2.3.3. Particle size distribution and particle radius were compared after 0 and 3 months of storage at 4–8 °C.

To evaluate therapeutic stability upon particle storage, the cytotoxic efficacy in cell culture was tested with the human adrenocortical carcinoma cell line NCI-H295R. Cells were seeded in a 2D monolayer and the CellTiter-Glo^®^ assay was performed 24 h after treatment with BSA-MT and DOPC-MT as described below. The assay was performed directly after NP preparation and repeated after 3 and 6 months of NP storage at 4–8 °C and 20–24 °C, respectively.

#### 2.4.2. pH Stability

For evaluating the influence of pH on the developed mitotane formulations (for explanation see Section 4), a pH stability study was performed. Particle size and zeta potential were measured as described in Section 2.3.1, using purified water and dispersion medium for sample dilution of BSA-MT and DOPC-MT, respectively. The pH was adjusted manually with 0.1 M HCl and 0.1 M NaOH to values between 2–10.

### 2.5. Cell Culture Studies

#### 2.5.1. Cultivation of NCI-H295R and GM00038 Cells

NCI-H295R cells were cultivated in DMEM/F12 containing 2% (*v*/*v*) Ultroser^®^ G and 1% (*v*/*v*) ITS. For GM00038 cultivation, Minimum Essential Medium Eagle was supplemented with 2.2 g/L NaHCO_3_ and 15% (*v*/*v*) FCS. Both cell lines were incubated at 37 °C, 5% CO_2_, and 95% humidity in 75 cm^2^ tissue culture flasks and split after reaching 80–90% confluency. All cells were free of mycoplasma.

#### 2.5.2. NCI-H295R 3D Spheroid Preparation

Adrenocortical carcinoma MCTS were generated by the liquid overlay method [26]. Briefly, 50 µL of an autoclaved and thereby liquefied 1.5% (*w*/*v*) agarose-PBS solution was aseptically added to each well of a 96-well microtiter plate. After cooling down for at least 30 min, 8000 cells per well were seeded in a volume of 200 µL. The plates were then centrifuged at 600× *g* and 20 °C for 5 min (Centrifuge 5804 R, Eppendorf AG, Hamburg, Germany) and incubated for 4 days until reaching a spheroid diameter of approximately 475 µm and circularity of 0.9. Circularity was calculated using ImageJ software as previously described [32]. Spheroid formation and growth were monitored using an Axiovert 40 CFL microscope with an Axiocam 305 color camera and a ZEN core v3.0 software (Carl Zeiss, Oberkochen, Germany).

#### 2.5.3. Cytotoxicity and Tolerability in Cell Culture

Cytotoxicity of the developed formulations was tested in NCI-H295R 2D monolayers (for therapeutic stability evaluation) and NCI-H295R 3D tumor spheroids, and tolerability was evaluated in GM00038 fibroblast 2D monolayers. All experiments were performed in white opaque 96-well microtiter plates. 

For the 2D monolayer experiments, NCI-H295R, as well as GM00038, were seeded at a density of either 20,000 or 3000 cells per well, respectively, 24 h prior to the treatment with 1–200 µM MT, DOPC-MT, or BSA-MT. After incubation for 24 h or 72 h, CellTiter-Glo^®^ luminescent cell viability assay was used according to the manufacturer’s protocol.

Adrenocortical carcinoma (NCI-H295R) 3D spheroids were prepared as described above and after 4 days of growth treated with 1–200 µM MT, DOPC-MT, or BSA-MT. Following an incubation time of 24 h and 72 h, CellTiter-Glo^®^ 3D cell viability assay was performed according to the manufacturer’s protocol. Cell viability was calculated as a percentage of untreated control cells. Additionally, BSA and empty DOPC liposomes were tested on both cell lines as a control for the carrier’s tolerability.

#### 2.5.4. Live/Dead Staining of NCI-H295R 3D Spheroids

NCI-H295R MCTS were prepared as described in Section 2.5.2 and treated with 5, 20, 75, 140, and 200 µM MT, BSA-MT, or DOPC-MT. After 72 h of incubation, spheroids were washed 3 times with PBS and subsequently stained with 5 µM calcein-AM and propidium iodide in PBS for 2 h at 37 °C. Living and dead cells were visualized with the Axiovert 40 CFL, using a 5 × objective and a corresponding filter set for calcein-AM and propidium iodide.

### 2.6. Statistics

All experiments were carried out at least in triplicate and results are expressed as mean ± standard deviation. Statistical analyses were carried out with GraphPad Prism 8.01 (GraphPad Software, San Diego, CA, USA). Differences between the mean of each sample and the mean of a control (unpaired) were evaluated by two-tailed Student’s *t*-test in the case of two different groups and by one-way ANOVA with Dunnett’s multiple comparison test when there were more than two different groups. Statistical differences were considered significant at *p* values < 0.05 (* < 0.05, ** < 0.01, *** < 0.001).

## 3. Results

### 3.1. Characterization and Stability of Nanoparticles

#### 3.1.1. Nanoparticle Preparation and Physicochemical Characterization

For the preparation of MT formulations, dual centrifugation as a closed vial system was used to enable simple handling of the cytostatic drug. Although this method is already established for the manufacturing of liposomes, it represents a novel approach for the preparation of albumin-stabilized NPs. The resulting BSA-MT NPs had a hydrodynamic diameter of 359 ± 7 nm, a PDI of 0.14 ± 0.03, indicating a narrow size distribution, a pH value of 6.5 ± 0.1, a zeta potential of −48 ± 6 mV, and drug content of 2.92 ± 0.23 mg/mL (Table 1). As BSA-MT has to be purified, the yield of the final NP suspension was determined as described in Section 2.3.2, resulting in a mean value of 61.9 ± 2.8%. The DOPC-MT liposomes, representing the second formulation strategy, exhibited a smaller hydrodynamic diameter of 117 ± 5 nm, a PDI of 0.20 ± 0.02, a pH value of 7.4 ± 0.1, and a zeta potential of −4 ± 1 mV (Table 1). HPLC analysis of MT and DOPC content resulted in a drug-to-lipid ratio of 0.1 ± 0.0 mol/mol, with a MT concentration of 0.67 ± 0.01 mg/mL.

#### 3.1.2. Asymmetric Flow Field-Flow Fractionation (AF4)

**Figure 1:**
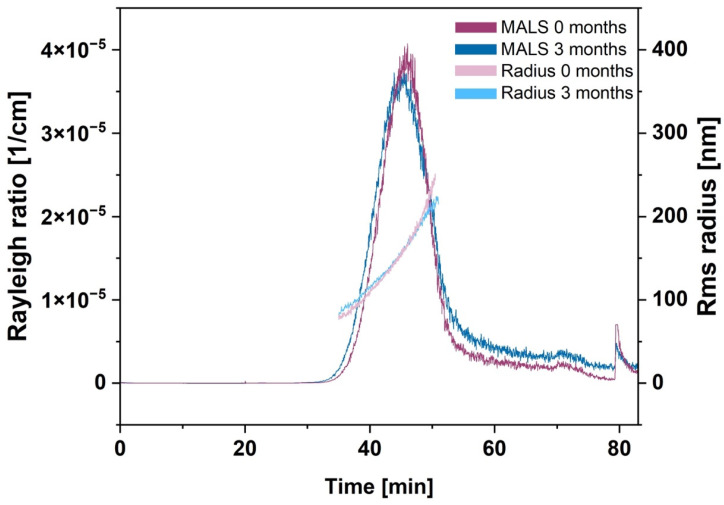
AF4 elugram of albumin-stabilized mitotane (BSA-MT) with MALS signal (90° angle) and corresponding rms radius. Particles were analyzed subsequently after preparation (0 months, purple) and 3 months of storage at 20–24 °C (blue).

AF4 measurements were performed to receive more detailed information about particle size distribution and particle populations of BSA-MT. The separation method was optimized for the maximal resolution of the NP sizes. Figure 1 shows a typical AF4 elugram with a MALS detector signal (90° angle), and the overlaid root mean square radii in an elution time frame from 35–50 min. Lower particle concentrations prohibited a precise radius calculation beyond this time frame. In addition to the large BSA-MT peak, unbound mono- and dimeric BSA could be detected at about 20 min (known from a preliminary experiment with a separate analysis of BSA), however, this peak was negligible. The MALS signal revealed the highest NP concentration at a retention time of about 45 min and an intensity-weighted mean rms radius of 193 ± 11 nm.

#### 3.1.3. Stability Studies

**Figure 2:**
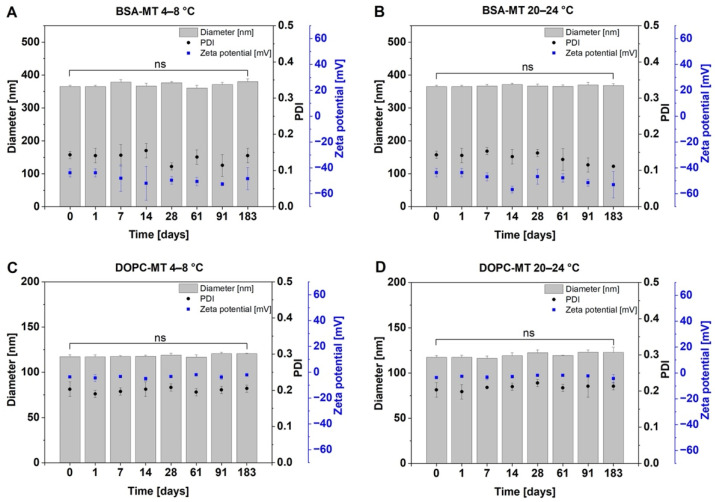
Colloidal stability analysis of albumin-stabilized mitotane (BSA-MT) and liposomal mitotane (DOPC-MT) under different storage conditions. Aliquots of the samples were stored at 4–8 °C ((**A**) BSA-MT; (**C**) DOPC-MT) or 20–24 °C ((**B**) BSA-MT; (**D**) DOPC-MT) for 6 months (183 days) and then analyzed for particle size, polydispersity index (PDI), and zeta potential. Significant differences were evaluated via one-way ANOVA and Dunnett’s post-hoc test (ns = not significant). Data are expressed as mean ± SD, *n* = 3.

**Figure 3:**
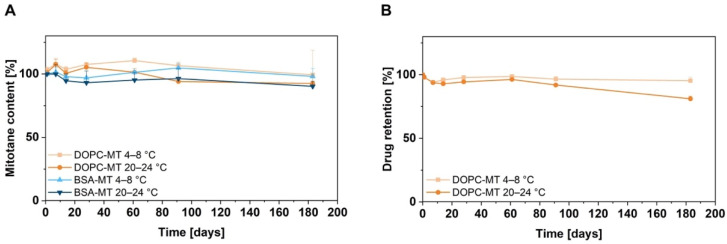
Stability analysis of the mitotane content of albumin-stabilized mitotane (BSA-MT) and liposomal mitotane (DOPC-MT) under different storage conditions. Aliquots of the samples were stored at 4–8 °C or 20–24 °C for 6 months (183 days) and analyzed for their mitotane content. (**A**) Mitotane content given in % of the initial drug content. (**B**) Drug retention of DOPC-MT. A value of 100% indicates no change in drug-to-lipid ratio and thus no drug leakage from liposomes. Data are expressed as mean ± SD, *n* = 3.

Storage stability is a crucial parameter in formulation development, as it can affect the efficacy and toxicity of the NPs, and determines the shelf life of the drug product. Thus, appropriate storage conditions have to be evaluated for each novel drug formulation. The colloidal storage stability of BSA-MT and DOPC-MT was investigated by measuring particle size, size distribution, and zeta potential at different storage temperatures over a 6-month period. As shown in Figure 2, no significant changes could be observed during 6 months of storage at 4–8 °C and 20–24 °C for both NP formulations (*p* > 0.05). In addition, mitotane content was analyzed as an indicator of drug degradation or formulation instability, revealing a constant drug content for both BSA-MT and DOPC-MT (Figure 3A; *p* > 0.05). For liposomes, a decreasing drug-to-lipid ratio during storage is a surrogate for drug leakage. Therefore, liposomes were separated from non-associated MT and analyzed as described in Section 2.4.1 for calculating the final drug retention. After 6 months, drug retention was still 95 ± 3% when stored at 4–8 °C, however, it decreased significantly to 81 ± 2% at 20–24 °C (Figure 3B, *p* = 0.0144).

Further stability studies of BSA-MT were performed with a focus on storage conditions at 4–8 °C. The AF4 analysis as described in Section 3.1.2 was repeated after 3 months of storage. Figure 1 shows the overlaid elugrams of BSA-MT analyzed after preparation (purple) and after storage (blue) and reveals equivalent particle size distributions, as well as particle radii for the NP peak. The intensity-weighted mean radius after 3 months was 186 ± 2 nm and, therefore, comparable to the radius after preparation (193 ± 11 nm, *p* > 0.05). Furthermore, a Bradford assay was performed to identify non-particulate albumin and evaluate NP stability based on BSA detached from the particles. Following NP centrifugation and separation from the supernatant, 59 ± 25 µg/mL and 57 ± 24 µg/mL albumin were detected after 0 and 3 months, respectively. Overall, the amount of non-particulate albumin was very low and did not change significantly upon particle storage (*p* > 0.05).

**Figure 4:**
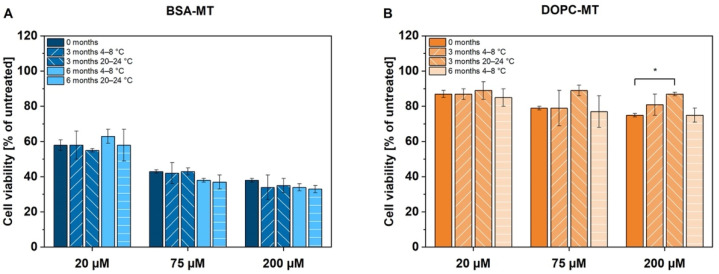
Therapeutic stability analysis of (**A**) albumin-stabilized mitotane (BSA-MT) and (**B**) liposomal mitotane (DOPC-MT) under different storage conditions. Cell viability of NCI-H295R cells was analyzed by the CellTiter-Glo^®^ luminescent cell viability assay 24 h after treatment with freshly prepared particles and particles stored for 3 and 6 months at 4–8 °C and 20–24 °C, respectively. Significant differences were evaluated via one-way ANOVA and Dunnett’s post-hoc test (* *p* < 0.05, further differences were not significant). Data are expressed as mean ± SD, *n* = 3.

For a final assessment of NP storage, therapeutic stability was analyzed using the ACC cell line NCI-H295R. Cells were treated with previously manufactured samples and cell viability was determined 24 h after treatment by CellTiter-Glo^®^ assay. The experiment was repeated after 3 and 6 months of particle storage. Cell viability was decreased to 38 ± 1% (0 months), 34 ± 7% (3 months, 4–8 °C), 35 ± 4% (3 months, 20–24 °C), 34 ± 2% (6 months, 4–8 °C), and 33 ± 2% (6 months, 20–24 °C), respectively, at a concentration of 200 µM BSA-MT (Figure 4A). No significant difference could be observed between the storage conditions (*p* > 0.05). However, 200 µM DOPC-MT decreased cell viability to only 75 ± 1% (0 months), 81 ± 6% (3 months, 4–8 °C) and 75 ± 4% (6 months, 4–8 °C; Figure 4B). Particle storage at 20–24 °C led to significantly reduced toxicity in ACC cells, with resulting viability of 87 ± 1% (*p* = 0.0208) and was thus finished after 3 months.

**Figure 5:**
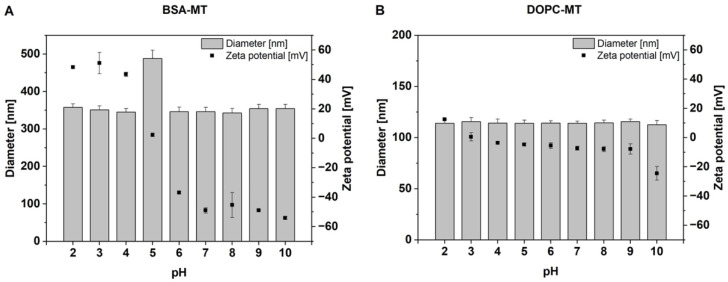
pH-dependent analysis of particle diameter and zeta potential of (**A**) albumin-stabilized mitotane (BSA-MT) and (**B**) liposomal mitotane (DOPC-MT). Data are expressed as mean ± SD, *n* = 3.

In addition to storage stability, the pH-dependent colloidal stability of the NP was also evaluated. In the range from pH 2–10, the hydrodynamic diameter of DOPC-MT was about 115 nm for all of the values tested (Figure 5B). However, the zeta potential showed a pH-dependent increase toward lower pH values and a decrease toward higher pH values, leading to a maximum of 13 ± 1 mV at pH 2 and a minimum of −25 ± 5 mV at pH 10. In contrast, the hydrodynamic diameter of BSA-MT was about 350 nm for all measured pH values, except pH 5, where a significant increase of about 140 nm was observed (Figure 5A, *p* < 0.0001). The zeta potential changed from negative values above pH 5 to positive values at the lower pH and covered a range of approximately −50 mV to 50 mV. Assuming a linear correlation between pH values in the range from 4–6 and zeta potential, an isoelectric point (IEP) of 5.0 could be determined.

### 3.2. Cell Culture Studies

#### 3.2.1. Cytotoxicity to NCI-H295R 3D Spheroids

ACC 3D spheroids were analyzed regarding their diameter and circularity over a period of 18 days (Figure S1). Although spheroid size increased continuously over time, circularity reached its maximum on day 4 (0.90 ± 0.02) and remained constant until day 15 when it decreased to 0.88 ± 0.01. This decline was caused by the formation of bulges at the rims of the spheroids, leading to inhomogeneous changes in shape. For optimal reproducibility, the following experiments were thus started on day 4 after cell seeding.

**Figure 6:**
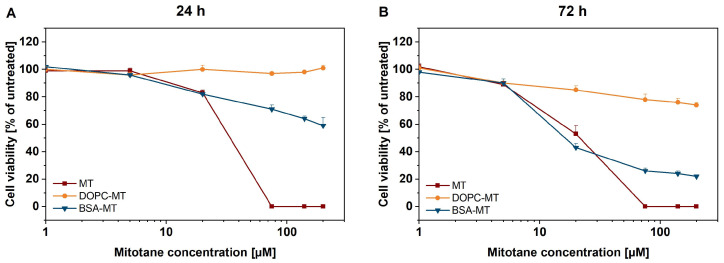
Representation of 3D tumor spheroids: in vitro cytotoxicity of albumin-stabilized mitotane (BSA-MT), liposomal mitotane (DOPC-MT), and free mitotane (MT). Cell viability of NCI-H295R 3D multicellular tumor spheroids was analyzed using the CellTiter-Glo^®^ 3D cell viability assay (**A**) 24 h and (**B**) 72 h after sample treatment. Data are expressed as mean ± SD, *n* = 3.

The cytotoxic potency of the developed NP formulations was evaluated using the CellTiter-Glo^®^ 3D cell viability assay. After an incubation period of 24 h, 200 µM BSA-MT decreased the viability to 59 ± 6%, whereas similar DOPC-MT concentration showed no cytotoxic effect (101 ± 2%; Figure 6A). The free MT control led to a complete decrease in cell viability when applied at a concentration of at least 75 µM. Notably, BSA-MT and free MT resulted in similar cytotoxicity at a concentration of 20 µM (approx. 80% viability). After 72 h of incubation, the BSA-MT cytotoxicity was even more pronounced (Figure 6B). The cell viability after treatment with 20 µM BSA-MT was in a range between 40–50% and was again comparable to that of free MT. Furthermore, at the highest-tested concentration of 200 µM BSA-MT, viability decreased to 22 ± 1%. However, DOPC-MT had little effect on NCI-H295R spheroids, leading to a viability of 74 ± 2% after treatment with 200 µM of the formulation. To exclude any cytotoxic effect caused by the NP carriers, experiments were also performed with unloaded DOPC liposomes and BSA in a broad concentration range (Figure S2). Although no effect could be observed for BSA, DOPC induced a moderate increase in cell viability.

**Figure 7:**
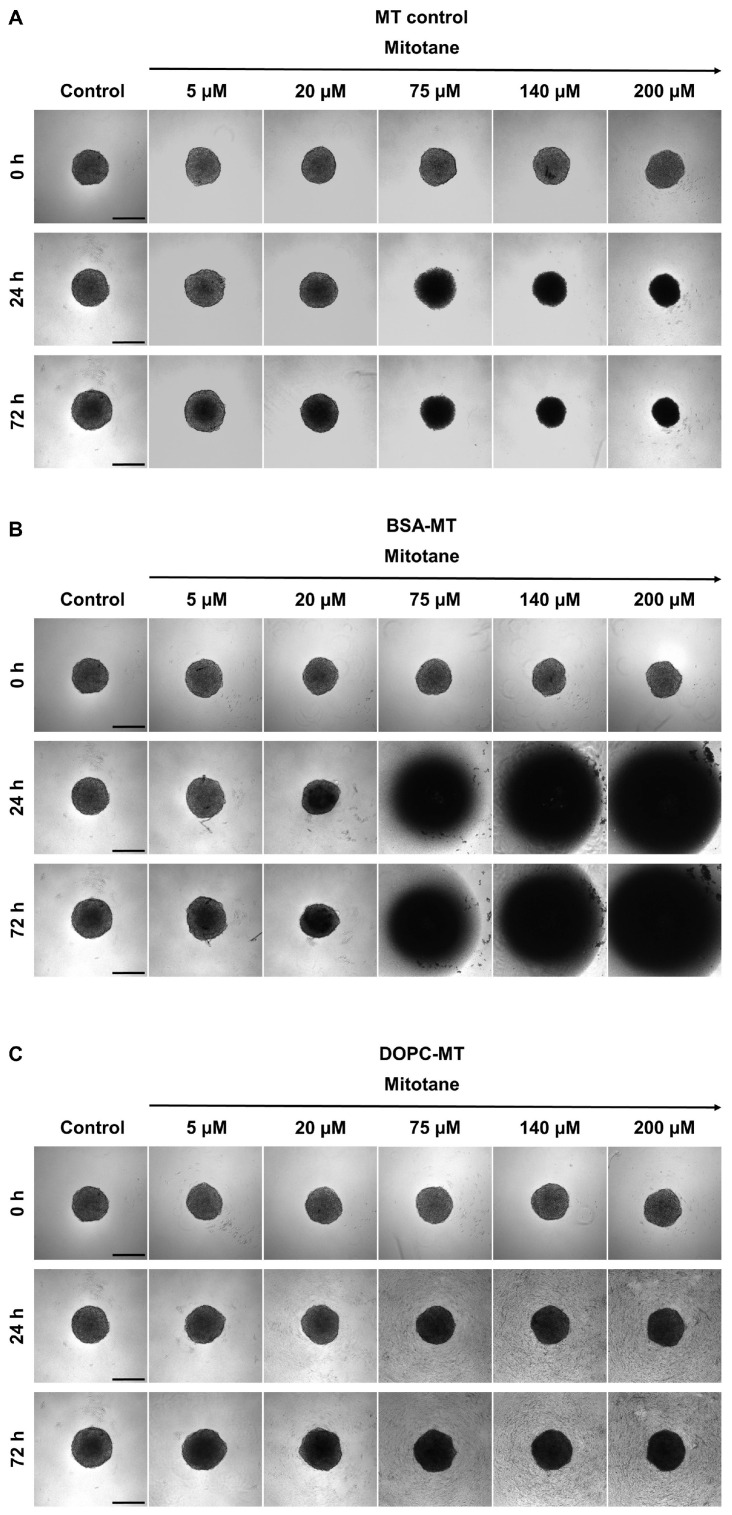
Representative brightfield microscopy images of NCI-H295R 3D multicellular tumor spheroids treated with (**A**) free mitotane (MT), (**B**) albumin-stabilized mitotane (BSA-MT), and (**C**) liposomal mitotane (DOPC-MT). Images were taken before treatment and after an incubation period of 24 h and 72 h. Scale bar = 400 µm.

Additionally, microscopic images were taken over time during treatment with NP formulations and MT. The incubation with free MT resulted in a reduction in spheroid growth, dependent on drug concentration and time, however, with preservation of the spherical shape (Figure 7A). The same effect could be observed for BSA-MT up to a concentration of 20 µM. Surprisingly, higher concentrations led to a complete disintegration of the spheroids (Figure 7B). In accordance with the results of the cell viability assay, DOPC-MT only slightly impacted spheroid growth (Figure 7C).

#### 3.2.2. Live/Dead Staining of NCI-H295R 3D Spheroids

**Figure 8:**
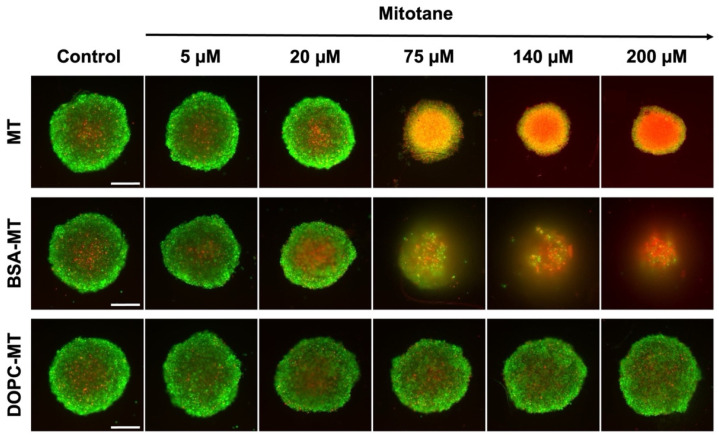
Representative fluorescence microscopy images of NCI-H295R 3D multicellular tumor spheroids based on live/dead staining. Spheroids were treated with free mitotane (MT), albumin-stabilized mitotane (BSA-MT), or liposomal mitotane (DOPC-MT), followed by an incubation period of 72 h. Cells were stained with a combination of calcein-AM (5 µM, green) and propidium iodide (5 µM, red) to visualize viable and dead cells, respectively. Scale bar = 200 µm.

For distinguishing between live and dead cells, NCI-H295R MCTS were stained with calcein-AM and propidium iodide for the last 2 h of a 72 h incubation period with the MT formulations. The untreated control was stained the same way and revealed the typical structure of tight, dense spheroids with a necrotic core (red) surrounded by proliferating and viable cells in the outer rim (green; Figure 8). This structure was changed after the treatment with free MT. At a concentration of 75 µM, spheroids consisted almost entirely of dead cells, with the propidium iodide signal being further intensified with increasing drug concentration. BSA-MT led to a comparable dose-dependent increase in dead cells, however, spheroid size was further decreased. At drug concentrations of at least 75 µM, the tight and dense spheroid structure disintegrated. Due to the washing steps related to the staining procedure, cells that were detached during spheroid disintegration (refer to brightfield microscopy; Figure 7B) were washed away. In contrast, no effect on the spheroid size, structure, or the number of dead cells could be observed with the DOPC-MT treatment, regardless of the concentration used.

#### 3.2.3. Tolerability in GM00038 Cells

**Figure 9:**
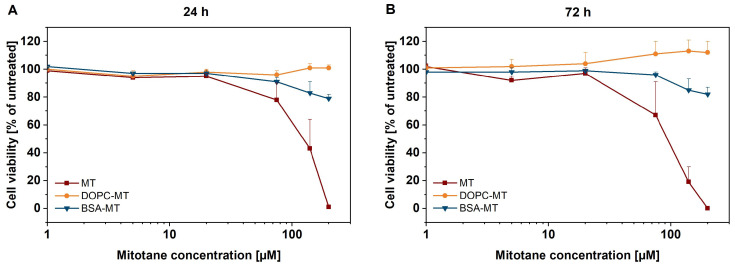
Representation of 2D cell culture: in vitro cytotoxicity of albumin-stabilized mitotane (BSA-MT), liposomal mitotane (DOPC-MT), and free mitotane (MT). Cell viability of GM00038 human dermal fibroblasts in a 2D monolayer model was analyzed using the CellTiter-Glo^®^ cell viability assay (**A**) 24 h and (**B**) 72 h after sample treatment. Data are expressed as mean ± SD, *n* = 3.

After evaluating NP efficacy in the ACC spheroid tumor model, tolerability in non-malignant human dermal fibroblasts (GM00038) was investigated. Cells were seeded in a 2D monolayer and treated with MT formulations at the same concentrations which were used for the malignant cells. Following an incubation period of 24 h, the CellTiter-Glo^®^ luminescent cell viability assay revealed dose-dependent cytotoxicity of free MT starting at 75 µM and leading to a complete decrease in cell viability at 200 µM (Figure 9A). In contrast, BSA-MT applied at the highest drug concentration resulted in remaining cell viability of 79 ± 3%. An extension of the incubation period to 72 h led to further increased cytotoxicity of free MT in the concentration range between 75–200 µM (Figure 9B). Similar to the well-tolerated BSA-MT treatment after 24 h, viability also remained stable after 72 h (200 µM BSA-MT), 82 ± 5% viability). No cytotoxic effect could be observed for DOPC-MT, regardless of drug concentration or incubation time. Controls were performed with empty carriers and showed results comparable to those achieved with the tumor spheroids (no effect of BSA, slight increase in viability with DOPC liposomes after 72 h incubation, Figure S3).

## 4. Discussion

In the first part of the presented study, novel nanoparticulate carrier systems for MT were prepared by dual centrifugation and subsequently analyzed with regard to their physicochemical characteristics and storage stability. In the second part of the study, the therapeutic potential of the developed formulations was compared in an ACC spheroid model and non-malignant fibroblasts.

Dual centrifugation was used as a well-known method for liposomal MT formulation (DOPC-MT). We could furthermore establish its use for the preparation of albumin-stabilized NPs (BSA-MT). The combination of the simple and time-saving method with the closed vial technology is a promising approach for formulation development and manufacturing of cytostatic drug products. As it turns out, BSA-MT could be prepared with a smaller size distribution than DOPC-MT. However, the hydrodynamic diameter of the BSA-MT formulation was larger than that of DOPC-MT. Preliminary experiments showed that a significant decrease in BSA-MT particle size is also achievable and should be further investigated. Glutaraldehyde is a widely used crosslinker for amino groups during albumin NP preparation in general, but the toxicological potential of its free form requires its elimination from the final NP suspensions by purification [33]. As a consequence of the glutaraldehyde purification, BSA-MT is characterized by a narrow particle size distribution, as well as by the minimal amount of unbound mono- and dimeric BSA detected by Bradford assay and reconfirmed by AF4 analysis. Nevertheless, even small BSA-MT particles remained in the discarded centrifugation supernatant, leading to a decreased NP yield. In comparison to BSA-MT, no purification is required for DOPC-MT liposomes. As DOPC-MT was prepared with 10 mol% MT (in relation to the lipid amount), the determined drug-to-lipid ratio indicates complete drug incorporation. 

The zeta potential of NPs is often used to evaluate colloidal stability, with a value of more than ±30 mV being classified as highly stable [34]. As the pH value of blood is about 7.4, but tumor tissue and intracellular compartments like lysosomes are characterized by lower pH [35], it is of crucial importance to determine NP stability over a broad pH range. The zeta potential of the MT formulations is mainly determined by the properties of DOPC and BSA. The phosphocholine structure of the neutral lipid leads to a predominantly pH-independent liposomal structure exhibiting a marginally negative zeta potential in the pH range of 4–9. In very acidic and alkaline conditions, zeta potential is influenced by protonation and deprotonation of the lipid, respectively, and hence a slight increase or decrease could be observed. Due to the neutral DOPC structure, liposome size was not affected by changes in pH value. In contrast, the zeta potential of BSA-MT was clearly negative (below −40 mV) at neutral and more alkaline pH values, indicating high colloidal stability. The zeta potential changes to zero at pH 5 and increases to about plus 40 mV in more acidic conditions. The change from a negative value to a positive value specifies the isoelectric point (IEP) of the NP formulation and correlates with the IEP of soluble albumin (approx. 4.7–5.3). Higher pH values cause a negative surface charge and thus zeta potential of the NP, whereas a positive surface charge and zeta potential occur at lower pH values [36]. At the IEP, electrostatic stabilization of the colloidal system is minimal, which might result in particle agglomeration [34]. This instability was indeed observed by an increase in particle size of about 140 nm at pH 5.

In addition to pH-dependent stability, the long-term storage stability of the NP was investigated at 4–8 °C and 20–24 °C. The effects of physical instability such as NP aggregation or fusion, especially of the liposomes, can be monitored by changes in average particle size and size distribution [37]. Albumin in particular is known for its NP-stabilizing function by adsorbing onto its surface, providing charge and steric stabilization, and thereby preventing particle aggregation [38]. This effect was visible for BSA-MT, as neither particle size nor homogeneity (PDI) was significantly altered upon 6 months of storage at 4–8 °C and 20–24 °C, respectively. Moreover, the constant MT drug content revealed no degradation of the active ingredient. For liposome evaluation, the loss of the associated drug (either entrapped or incorporated) caused by leakage is an additional critical parameter. Even though DOPC-MT exhibited colloidal stability at both storage temperature ranges, the drug retention decreased significantly during storage at 20–24 °C after 6 months. The observed drug loss from DOPC-MT liposomes was also confirmed by a decrease in cytotoxic activity in ACC cells. Although the CellTiter-Glo^®^ assay revealed a constant decrease in cell viability by BSA-MT, regardless of the storage condition, a significant increase in cell viability (decrease in cytotoxic effect) was observed for 200 µM DOPC-MT after 3 months of storage at 20–24 °C. This loss of therapeutic activity can be explained by leakage of MT from the liposomes, leading to the drug being unavailable for the tumor cells. Therefore, the optimal storage conditions were identified as 4–8 °C, which is in agreement with the recommendation to store liposomes at 4–6 °C in order to minimize the risk of temperature-dependent phospholipid hydrolysis [37].

Further stability experiments with AF4 were performed solely with BSA-MT because it was the most promising formulation. The adsorption of BSA-MT to the separation membrane in the AF4 channel required multiple NP injections prior to analysis to achieve membrane saturation. In the following, the AF4 elugram revealed an approximately symmetric NP peak. The slightly delayed decline of the peak indicated a small fraction of bigger NP with a broader size distribution. However, through the results of the dynamic light scattering (PCS) measurements, this NP peak remained unaltered after 3 months of particle storage, thus confirming the particle-stabilizing effect of albumin. As the intensity-weighted mean rms radius of the NP peak and the hydrodynamic diameter determined by PCS are based on different calculation models, their values are not comparable without restrictions. However, the measurement of similar particle sizes could be regarded as confirmation that PCS (as the more straightforward approach) is a suitable analytical method for routine size determination of BSA-MT. For quantifying the small peak of non-particulate BSA assumed in the elugram, a Bradford assay was carried out at the same time as the AF4 analysis. As expected, the amount of unbound BSA was negligible due to the purification step during the initial particle preparation. The calculated SD indicates differences among the analyzed samples, yet the detected albumin amount in each sample remained unchanged between 0 and 3 months of storage. Albumin was therefore not detached from BSA-MT during particle storage, again demonstrating a continuous stabilization of the drug NP. The variations among the samples were mainly due to the manual purification process. For future optimization of NP preparation, automation of the process could be beneficial. Additionally, future studies should be performed investigating further impacts (e.g., oxygen, light, dilution) on NP stability and degradation. Depending on the results, a lyophilization step comparable to Abraxane^®^ could also be taken into consideration for long-term storage. With regard to upcoming in vivo experiments, it is also recommended that the serum stability of BSA-MT be analyzed. 

In the second part of this study, the in vitro cytotoxicity of the developed NPs to malignant NCI-H295R (2D and 3D cell culture models) and the tolerability in non-malignant GM00038 fibroblasts were evaluated. Both BSA-MT and DOPC-MT affected NCI-H295R 3D spheroid viability in a time- and concentration-dependent manner. However, in contrast to DOPC-MT, BSA-MT could decrease MCTS viability more effectively and comparably to the free drug after an incubation period of 72 h. Noticeably, MT is mainly bound to plasma lipoproteins in vivo, making it pharmacologically inactive [9,10], suggesting that the free drug in vitro could lead to an overestimation of cytotoxicity compared to similar NP concentrations. A future study comparing the lipoprotein binding of BSA-MT and MT would be advisable to further investigate the therapeutic potential of BSA-MT.

Compared to the albumin-stabilized NPs, the liposomal MT formulation showed low cytotoxicity in the ACC 3D spheroid model. Since MT acts mainly intracellularly by affecting adrenal mitochondria [39], cellular uptake becomes a key factor in NP efficacy and is thereby likely a factor in the poor potency of DOPC-MT. One aspect crucial for tissue penetration and efficient uptake is the size of a nanocarrier [40,41]. Nanomaterials for drug delivery are often associated with dimensions of less than 100 nm, however, recent publications challenge the wisdom that “the smaller the better” [42,43]. It has been reported that, depending on the cell line, NPs with 325 nm and 220 nm size led to a higher cellular uptake compared to smaller particles [43]. Therefore, BSA-MT could potentially be advantageous compared to DOPC-MT for ACC cell uptake. The second important aspect is the specific uptake mechanism, depending on the nanocarrier. Although distinct mechanisms have not been conclusively defined and are currently the focus of multiple studies, it has been shown that albumin-stabilized drug nanoparticles are characterized by high cellular uptake [44,45]. It is hypothesized that albumin-mediated transcytosis allows them to cross endothelial cells and reach tumors [46]. The subsequent tumor accumulation is presumably enhanced by the presence of albumin-binding proteins such as the “secreted protein acidic and rich in cysteine” (SPARC) found in the tumor microenvironment. Compared to healthy tissue, a higher prevalence of SPARC was found in the interstitium of various tumors, including ACC [22,47]. A similar strategy for increasing liposome uptake by tumors could be active targeting. Since ACC is also characterized by an overexpression of the insulin-like growth factor 1 receptor (IGF1-R), anti-IGF1-R immunoliposomes have been described as an effective approach for enhancing cellular uptake and should therefore be investigated in future studies for improving the formulation of DOPC-MT [48,49].

The superiority of BSA-MT over DOPC-MT was also evident in microscopy studies because the liposome formulation showed no influence on spheroid shape or cell viability. On the contrary, the free drug and the albumin-stabilized drug NPs resulted in a significant increase in dead cells, with the latter also leading to a disintegration of the spheroids. Different mechanisms of inducing cell death could influence these cellular binding properties and in turn spheroid morphology. Apoptosis in particular leads to the loss of cell-cell contacts, and, therefore, differences in the induction of apoptosis and necrosis might be caused after treatment with MT and BSA-MT [50,51]. It has recently been shown that a combinational therapy of MT and nilotinib was more efficient compared with single MT treatment in decreasing NCI-H295R cell viability, similarly leading to distinct spheroid disintegration [52]. Therefore, the injection of BSA-MT could be beneficial in comparison with the current MT therapy regimen and lead to a significant improvement in ACC treatment. However, although MCTS represent a superior cell model compared with 2D cell experiments, further in vivo studies are required to evaluate the effect of NPs in humans. 

Anticancer drugs should not cause harm to non-malignant tissues in spite of their effectiveness against tumors. Since it was shown that patients tolerated higher drug doses of albumin-bound paclitaxel (Abraxane^®^) without an increase in toxicity [20,21], high concentrations of the developed MT formulations were evaluated for tolerability in GM00038 human dermal fibroblasts. Apparently, the toxicity of free MT was to some degree correlated with the therapy-limiting side effects of MT treatment which occurred at drug concentrations above 20 mg/mL (62 µM). In contrast, BSA-MT revealed significantly lower cytotoxicity, becoming particularly evident at the higher concentration of 200 µM, leading to the assumption that BSA-MT is more effective in tumor cells than DOPC-MT, while simultaneously being well-tolerated in non-tumor cells. Considering that NCI-H295R spheroids showed a pronounced BSA-MT sensitivity at this concentration, cell line-specific NP internalization is possible. As previously described, the uptake rate of NPs was found to be lower for non-malignant cells compared with cancer cells [53,54]. This particular effect for the BSA-MT formulation could remarkably improve ACC therapy with MT and should be investigated in future studies examining cellular uptake. Furthermore, investigations with additional ACC cell lines are advisable for gaining a deeper knowledge of BSA-MT efficacy with regard to future in vivo studies.

## 5. Conclusions

This study focuses on the comparative evaluation of two novel nanotechnological strategies for MT formulation based on liposomes and albumin-stabilized drug nanoparticles, respectively. Storage stability at 4–8 °C was proven for both formulations and in addition at 20–24 °C for BSA-MT. We were able to show that BSA-MT was significantly more effective in treating NCI-H295R 3D spheroids than DOPC-MT. This effectivity was accompanied by the desired tolerability in human dermal fibroblasts, which were used as a cell model for healthy tissue. According to these promising results, testing in an in vivo model would be the next step for evaluating the potential of BSA-MT in ACC treatment and should therefore be the focus of future studies.

## Figures and Tables

**Table 1:** Hydrodynamic diameter, polydispersity index (PDI), zeta potential, and mitotane content of albumin-stabilized mitotane (BSA-MT) and liposomal mitotane (DOPC-MT). Data are expressed as mean ± SD, *n* ≥ 10.

	Hydrodynamic Diameter [nm]	PDI	Zeta Potential [mV]	Mitotane Content [mg/mL]
**BSA-MT**	359 ± 7	0.14 ± 0.03	−48 ± 6	2.92 ± 0.23
**DOPC-MT**	117 ± 5	0.20 ± 0.02	−4 ± 1	0.67 ± 0.01

## Data Availability

Not applicable.

## Supplementary Materials

The following supporting information can be downloaded at: https://www.mdpi.com/article/10.3390/pharmaceutics14091891/s1, Figure S1: diameter and circularity of NCI-H295R multicellular tumor spheroids as a function of time; Figure S2: in vitro cytotoxicity of bovine serum albumin and unloaded DOPC liposomes to NCI-H295R 3D spheroids; Figure S3: in vitro cytotoxicity of bovine serum albumin and unloaded DOPC liposomes to human dermal fibroblasts (GM00038).
