# Supplementary material for: Mitotane Nanocarriers for the Treatment of Adrenocortical Carcinoma: Evaluation of Albumin-Stabilized Nanoparticles and Liposomes in a Preclinical In Vitro Study with 3D Spheroids
Source: Pharmaceutics. 2022 Sep 7;14(9):1891. doi: 10.3390/pharmaceutics14091891 (PMC9501383; doi:10.3390/pharmaceutics14091891)
Supplement: Supplementary file 1 [file pharmaceutics-14-01891-s001.zip › pharmaceutics-1842790-supplementary.pdf]

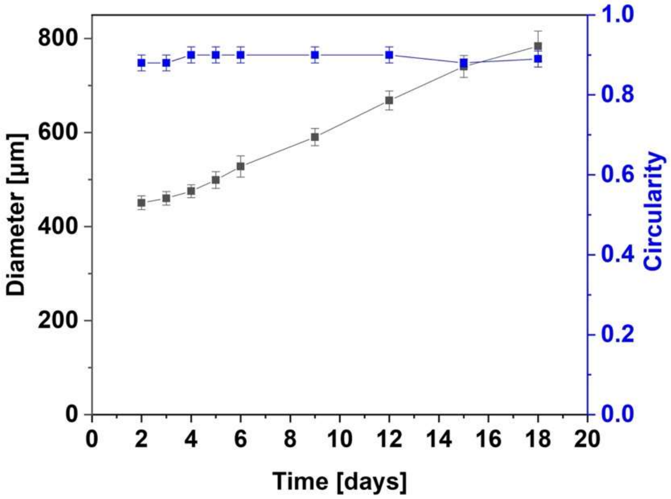

Figure S1: Diameter and circularity of NCI-H295R multicellular tumor spheroids as a function of time. Spheroids were prepared by liquid overlay method and microscopically analyzed in the following days after cell seeding (t = 0 days). Data are expressed as mean ± SD, *n* = 10.

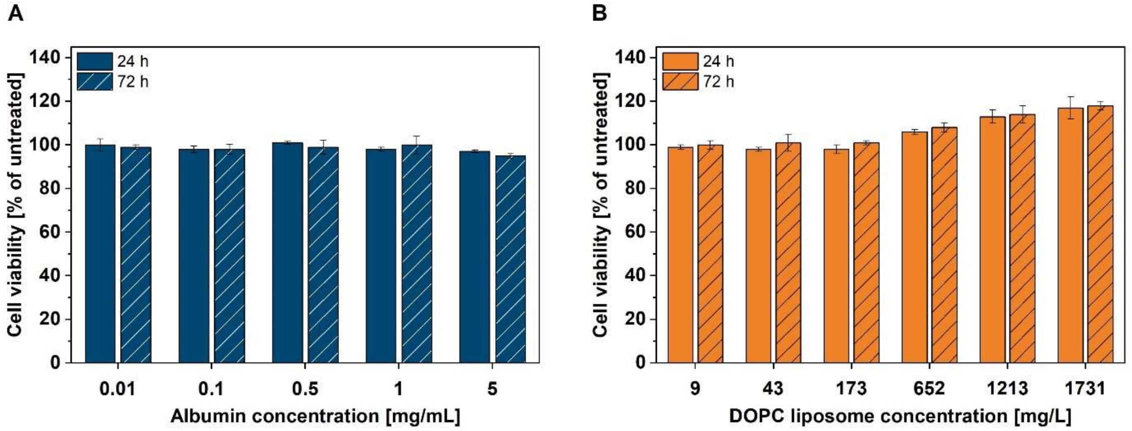

Figure S2: *In vitro* cytotoxicity of (**A**) bovine serum albumin and (**B**) unloaded DOPC liposomes. Cell viability of NCI-H295R 3D multicellular tumor spheroids was analyzed using CellTiter-Glo® 3D cell viability assay 24 h and 72 h after sample treatment. Data are expressed as mean ± SD, *n* = 3.

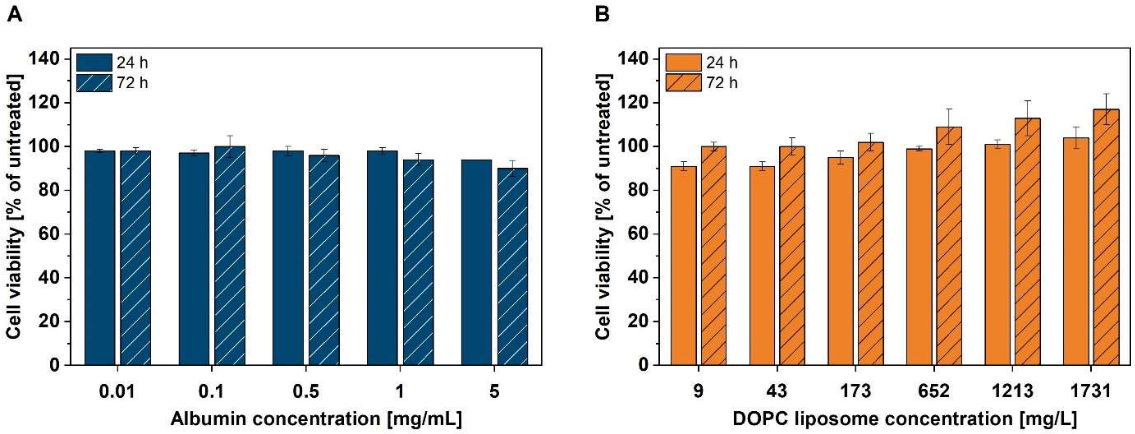

Figure S3: *In vitro* cytotoxicity of (**A**) bovine serum albumin and (**B**) unloaded DOPC liposomes. Cell viability of human dermal fibroblasts (GM00038) cultured as 2D monolayer was analyzed using CellTiter-Glo® cell viability assay 24 h and 72 h after sample treatment. Data are expressed as mean ± SD, $n = 3$.
